# Nitrogen fixation and *nifH* gene diversity in cyanobacteria living on feather mosses in a subalpine forest of Mt. Fuji

**DOI:** 10.1007/s00442-023-05334-9

**Published:** 2023-02-20

**Authors:** Masayuki Kubota, Norihisa Matsushita, Toshihiko Nakamura, Kenji Fukuda

**Affiliations:** Graduate School of Agricultural and Life Sciences, The University of Tokyo, Bunkyo-Ku, Tokyo, Japan

**Keywords:** Bryophyte, East Asia, Colonization, Community composition, Symbiosis

## Abstract

In the boreal forests, feather mosses such as *Hylocomium splendens* and *Pleurozium schreberi* are colonized by cyanobacteria, which provide large amounts of nitrogen to forest ecosystems through nitrogen fixation. Although these feather mosses are also ubiquitous in subalpine forests of East Asia, little is known regarding their associated cyanobacteria and their ability to fix nitrogen. In this study, we investigated (1) whether cyanobacteria co-exist and fix nitrogen in the two species of feather mosses that cover the ground surface in a subalpine forest of Mt. Fuji, (2) whether cyanobacteria belonging to a common cluster with boreal forests are found in feather mosses in Mt. Fuji, and (3) whether moss-associated nitrogen fixation rates differed among moss growing substrates, canopy openness, and moss nitrogen concentrations in the same forest area. Our results showed that cyanobacteria colonized feather mosses in the subalpine forests of Mt. Fuji and acetylene reduction rates as an index of nitrogen fixation tended to be higher in *H*. *splendens* than in *P*. *schreberi*. Based on analysis of the *nifH* gene, 43 bacterial operational taxonomic units (OTUs) were identified, 28 of which represented cyanobacteria. Among the five clusters of cyanobacteria classified based on their *nifH* gene and identified in northern Europe, four (*Nostoc* cluster I, *Nostoc* cluster II, *Stigonema* cluster, and *nifH*2 cluster) were also found at Mt. Fuji. The acetylene reduction rate differed depending on the moss growing substrate and the total nitrogen concentration of moss shoots, and a strong negative correlation was observed with the total nitrogen concentration.

## Introduction

Boreal forests are widely distributed in the Northern Hemisphere. Feather mosses cover the forest floor in these forests and have critical effects on soil moisture, temperature (Gornall et al. 2007), and chemistry (Cornelissen et al. 2007; Pacé et al. 2020). These feather mosses symbiotically associate with nitrogen-fixing cyanobacteria (DeLuca et al. 2002). The amount of nitrogen fixation by cyanobacteria associated with feather mosses is up to 7 kg N ha^−1^ year^−1^ (DeLuca et al. 2007; Lindo et al. 2013), which is comparable to or greater than the amount of nitrogen deposition in boreal forests (Gundale et al. 2011). Consequently, cyanobacteria on mosses play an important role in nitrogen cycling in boreal forests, where plant-available nitrogen is limited (Rousk et al. 2013).

Nitrogen fixation by free-living bacteria, including cyanobacteria, is often spatially heterogeneous, even within a single ecosystem component, and there are hotspots with particularly high fixation rates. In some cases, hotspots and non-hotspots are only a few centimeters apart (Reed et al. 2011). Moss-associated nitrogen fixation rates vary widely among and within forests (Deluca et al. 2002; Markham et al. 2009) and hotspots exist (Stuart et al. 2021b). Abiotic factors that affect the rate of nitrogen fixation by cyanobacteria on moss mats include water (Gundale et al. 2009, 2012b), temperature (Gundale et al. 2012a), light (Solheim et al. 2004; Gundale et al. 2012b), nitrogen availability (Zackrisson et al. 2004; Sorensen et al. 2012), and phosphorus availability (Zackrisson et al. 2004). Factors that cause variation in nitrogen fixation by cyanobacteria associated with feather mosses have been investigated in manipulative experiments. In addition, assessing the role of environmental factors in moss-associated nitrogen fixation by observational studies using natural gradients of nutrients or seasonal variations can validate the results of manipulation experiments in a real environment (Renaudin et al. 2022). However, few observational studies have addressed the environmental factors controlling spatial variation in nitrogen fixation within a single forest stand. The influence of the moss-growing substrate on moss-associated nitrogen fixation has also been overlooked. Decaying fallen logs promote seedling establishment in boreal and subalpine forests and are ecologically important in forest regeneration (Simard et al. 1998; Narukawa et al. 2003). Fallen logs can also be active sites for N_2_ fixers, mainly methanotrophs, and the acetylene reduction rate varies depending on the extent of their decay (Mäkipää et al. 2018). However, little is known about differences in moss-associated nitrogen fixation on fallen logs and on the ground.

Moss-associated nitrogen fixation is largely controlled by the taxonomic identity of mosses (Stuart et al. 2021b). Because the two feather moss species, *Hylocomium splendens* and *Pleurozium schreberi*, are ubiquitous in boreal forests, many studies have evaluated the relationship between nitrogen fixation and biotic factors in their communities. Both *H*. *splendens* and *P*. *schreberi* are typical boreal forest feather mosses, but *H*. *splendens* prefers more productive sites, whereas *P*. *schreberi* is distributed over a wider range of ecological conditions (Påhlsson 1994). Both *H*. *splendens* and *P*. *schreberi* decrease the nitrogen fixation rate per unit area of moss as N availability increases, but nitrogen fixation associated with *H*. *splendens* is tolerant to low amounts of N supply (5 kg N ha^−1^ year^−1^) (Zackrisson et al. 2009). Also, differences in hydration rates brought about by morphological characteristics of mosses in both species control cyanobacterial colonization and nitrogen fixation (Liu and Rousk 2022). *H*. *splendens* and *P*. *schreberi* are colonized by epiphytic cyanobacteria of the genera *Calothrix*, *Cylindrospermum*, *Nostoc*, and *Stigonema* (Gentili et al. 2005; Houle et al. 2006; Zackrisson et al. 2009; Ininbergs et al. 2011). A phylogenetic analysis based on the nitrogenase gene (*nifH*) identified five clusters of cyanobacteria that grow on *H. splendens* and *P. schreberi* in Swedish forests (*Nostoc* cluster I, *Nostoc* cluster II, *Stigonema* cluster, *nifH*2 cluster, and mixed cluster). That same study found evidence of a host-specific relationship between feather moss species and cyanobacterial communities (Ininbergs et al. 2011). Furthermore, changes in the expression of *nifH* in each of these clusters result in seasonal variations in nitrogen fixation associated with *H*. *splendens* and *P*. *schreberi* (Warshan et al. 2016). In Alaskan mosses (including *H*. *splendens* and *P*. *schreberi*), the bacterial community composition, including cyanobacteria, is also strongly host-specific (Holland-Moritz et al. 2018, 2021). There is a correlation between the phylogenetic distance of the host mosses and differences in the composition of the moss microbiome, and some putative N_2_-fixing bacteria other than cyanobacteria have their relative abundance correlated with the rate of nitrogen fixation associated with mosses (Holland-Moritz et al. 2021).

Coniferous forests consisting of *Abies* spp. and *Picea* spp., as well as boreal forests, are ubiquitous in the subalpine zone of the high mountains of East Asia; *Tsuga* spp. are also present (Franklin et al. 1979; Ohsawa 1990, 1993). In subalpine coniferous forests at altitudes of 1500–2500 m in central Honshu, Japan, communities of mosses common to boreal forests (e.g., *H. splendens* and *P. schreberi*) develop on the forest floor. In these settings, mosses often cover the entire forest floor, and the two aforementioned species constitute > 97% of the moss community (Nakamura 1984). By supporting seedling establishment, moss communities play an important role in the maintenance and regeneration of subalpine coniferous forests (Franklin et al. 1979; Nakamura 1987, 1992; Sugita and Nagaike 2005; Katsumata et al. 2008); a close relationship between mosses and nitrogen-fixing cyanobacterial communities might contribute to the maintenance and regeneration of these forests. However, neither nitrogen fixation by cyanobacteria, nor the community composition of cyanobacteria associated with the feather mosses *H. splendens* and *P. schreberi* in coniferous forests in East Asia (e.g., subalpine forests in Japan), has been extensively investigated.

This study was conducted in Japanese subalpine forests, especially in the subalpine forests of Mt. Fuji, where a homogeneous moss mat is spreading on the forest floor, and evaluated three hypotheses: (1) nitrogen fixation associated with *H*. *splendens* and *P*. *schreberi* occurs in the subalpine forests of Mt. Fuji, which is spatially distant from boreal forests in northern Europe and North America; (2) cyanobacteria in the same clusters as those in boreal forests are present in subalpine forests; and (3) moss-related nitrogen fixation varies with moss nitrogen demand, light conditions, and growth substrate, even within the same forest stand.

## Materials and methods

### Study site

The study site is a subalpine coniferous forest (35°23.9′ N, 138°42.0′ E, 2000 m above sea level) located on the north slope of Mt. Fuji. The study site is a natural forest located within a special zone (nature reserve area) of Fuji-Hakone-Izu National Park, where forest management and grazing are not conducted. The annual mean temperature of the study area is 4.4 °C, according to data from Japan Meteorological Agency stations located at the summit of Mt. Fuji and at Lake Kawaguchi. The mean annual precipitation near the study site is 2500 mm (Yamamoto 1971).

The surveyed forest is a climax forest (basal area 63.3 m^2^ ha^−1^), dominated by *Tsuga diversifolia* (relative basal area 98%) but interspersed with *Abies veitchii*, *Abies mariesii*, and *Rhododendron brachycarpum*. The forest floor is covered with well-mixed moss communities, dominated by *H*. *splendens* and *P*. *schreberi* (86% and 71% of vegetation cover, respectively), with a small proportion of *Dicranum majus* (5%).

### Sampling

Sampling was conducted on September 16, 2016. Ten shoots each of *H. splendens* and *P. schreberi* were collected from 10 fallen logs and from the ground surface within 1 m of each log in an approximately 1-ha area of the study site; thus, 400 moss shoots were collected in total (2 moss species × 2 habitats [log and ground] × 10 sampling locations × 10 shoots). The collected moss shoots were stored at 5–8 °C; nitrogen fixation rates were measured within 12 days.

### Measurement of acetylene reduction rate

To estimate the nitrogen fixation rates associated with the sampled mosses, acetylene reduction rates were measured according to the method of Hardy et al. (1968). Five shoots of *H. splendens* and 10 shoots of *P. schreberi*, sprayed with purified water at each sampling location and habitat, were enclosed in a 27 mL glass vial with a septum (Ininbergs et al. 2011). With the exception of the rhizoid, the entire shoot was used. The vials containing the moss shoots were incubated at 23 °C for 2 days under a light:dark cycle of 16 h light (photosynthetic photon flux density: 44 μmol m^−2^ s^−1^):8 h dark. After incubation, vials were ventilated and plugged. Next, 2.7 mL of air was removed from each vial and the same volume of acetylene gas was added. The samples were incubated under the same conditions for 1 day. After incubation, 1 mL of the gas phase in the vial was collected and the amount of ethylene produced was measured using a gas chromatograph (G-3500; Hitachi, Tokyo, Japan). The column packing material of the gas chromatograph was activated alumina, and a flame ionization detector was used. After the rate of cyanobacterial colonization had been measured, the shoots were freeze-dried, and their dry weights were determined. The amounts of ethylene produced by the natural reduction of acetylene (production of ethylene in the absence of a sample) and by the moss itself (production of ethylene by a sample without acetylene addition) were measured as controls; the total amount of ethylene in the samples minus the total amount of ethylene in the controls was regarded as the true amount of ethylene produced by the samples. Total ethylene production in the controls was calculated per vial. The acetylene reduction rate per shoot dry weight was calculated from the true amount of ethylene produced by the samples as an index of nitrogen fixation.

### Measurement of cyanobacterial colonization rate

After the nitrogen fixation rate had been measured, 10 shoots of each sample were observed under an epifluorescence microscope (SZX12; Olympus, Tokyo, Japan) fitted with a green excitation filter. The cyanobacterial colonization rate was calculated for each sample as the proportion of shoots containing cyanobacteria. For *H. splendens*, the cyanobacterial colonization rate was calculated by combining the five shoots used in the nitrogen fixation rate measurements with five other shoots collected from the same sampling location and habitat.

### Environmental factors that correlate with the rate of acetylene reduction and cyanobacterial colonization

Eco-physiological factors that correlate with acetylene reduction and cyanobacterial colonization on moss mats were assessed by measuring total nitrogen concentration in moss shoots and canopy openness at each sampling location for both *H*. *splendens* and *P*. *schreberi*. Canopy openness was determined in panoramic photographs that had been acquired at each sampling location using a 360° camera (THETA S; Richo, Tokyo, Japan); the openness was calculated using RGBFisheye (Ishida 2004). Samples used to determine the rates of nitrogen fixation and cyanobacterial colonization were homogenized at 1800 rpm for 90 s using a mixer mill (MM400; Verder Scientific, Haan, Germany). The total nitrogen concentrations in the samples were analyzed using an elemental analyzer (SUMIGRAPH NCH-22; Sumika Chemical Analysis Service, Tokyo, Japan).

### Examination of cyanobacteria community composition based on *nifH* gene analysis

The community composition of cyanobacteria living on each sample of *H. splendens* and *P. schreberi* was investigated via phylogenetic analysis of the *nifH* gene. DNA was extracted from 10 mg of the above-described homogenized moss leaf samples using the DNeasy Plant Mini Kit (Qiagen, Hilden, Germany).

A tenfold dilution of the DNA extract was used as the template; the *nifH* region of cyanobacterial DNA was polymerase chain reaction (PCR)-amplified using the cyanobacteria-specific primers CNF/CNR (Olson et al. 1998), together with the KAPA Taq EXtra PCR kit (KAPA Biosystems, Boston, MA, USA). The PCR solution was prepared in accordance with the kit manufacturer’s protocol. Amplification was performed as follows: initial denaturation at 95 °C for 3 min; 40 cycles of denaturation at 94 °C for 30 s, annealing at 55 °C for 30 s, and extension at 72 °C for 1 min; and final extension at 72 °C for 7 min.

PCR products were integrated into the pMD20 T-vector (Takara Bio, Shiga, Japan) using the DNA Mighty Mix ligation kit (Takara Bio), then transferred into *Escherichia coli* HST08 competent cells (Takara Bio) in accordance with the manufacturer’s instructions. The subcloned fragments were amplified from plasmid DNA in 12 positive clones using the primers U19 and M13R. The PCR products were purified using Illustra ExoProStar (GE Healthcare, Buckinghamshire, UK) and sequenced by Macrogen Japan (Tokyo, Japan) using primer SP6.

The obtained 480 sequences were clustered using CD-HIT Suite (http://weizhongli-lab.org/cdhit_suite/cgi-bin/index.cgi?cmd=cd-hit-est; Huang et al. 2010) and classified into operational taxonomic units (OTUs), using 97% sequence homology as the threshold. Representative sequences of each OTU were searched against the database of bacteria with high homology via BLAST (https://www.ncbi.nlm.nih.gov/) and divided into cyanobacteria or non-cyanobacteria according to the most homologous reference sequence. OTU sequences identified as cyanobacteria were combined with reference sequences that belonged to *Nostoc* cluster I, *Nostoc* cluster II, *Stigonema* cluster, *nifH*2 cluster, and mixed cluster (Ininbergs et al. 2011); multiple alignments were generated using MAFFT version 7 (https://mafft.cbrc.jp/alignment/server/; Kuraku et al. 2013; Katoh et al. 2019). A maximum likelihood phylogenetic tree was created using the IQ-TREE web server (http://iqtree.cibiv.univie.ac.at/; Nguyen et al. 2015; Trifinopoulos et al. 2016; Kalyaanamoorthy et al. 2017) with *Trichodesmium erythraeum* (Accession number L00689) as the outgroup. Bootstrap values were calculated from 1000 iterations of bootstrap resampling via UFBoot (Hoang et al. 2018). Reference sequence information was obtained from GenBank and from Integrated Microbial Genomes (http://img.jgi.doe.gov/). Sequences of each OTU are registered in the DDBJ database (accession numbers LC716857–LC716899).

### Statistical analysis

Differences in log-transformed acetylene reduction rates by moss species and moss growth substrate (fallen logs/ground) were examined by repeated measures analysis of variance (ANOVA) and post hoc analysis by Tukey's honestly significant difference test. The normality of the data was confirmed by the Shapiro–Wilk test and the homoscedasticity of each group by the Bartlett test. Multiple comparisons of cyanobacterial colonization rates were conducted by Bonferroni-corrected Wilcoxon signed-rank test between moss species and growth substrates. A simple linear regression analysis was performed with log-transformed acetylene reduction rate as the objective variable and cyanobacterial colonization rate as the explanatory variable.

A generalized linear model (GLM) was used to analyze environmental factors correlated with acetylene reduction rates and cyanobacterial colonization rates for each moss species. Growth substrate (fallen logs/ground), canopy openness of the sampling location, and total nitrogen concentration in moss shoots served as explanatory variables. In GLMs, a gamma error distribution and log link function were used for the acetylene reduction rate, while a pseudo-binomial error distribution and logit link function were used for the colonization rate. Hypothesis tests of the data fitted to the GLM were conducted using an analysis of deviance; *F* tests were employed to extract factors that correlate with the rates of acetylene reduction and cyanobacterial colonization.

A permutational multivariate analysis of variance (PERMANOVA) was conducted to evaluate differences in the cyanobacterial community composition according to moss species, growth habitat, and sampling location. The β-diversity index, which represents the difference in cyanobacterial community composition among samples, was determined using both the Bray–Curtis index, calculated using the number of confirmed cyanobacterial OTU sequences in each sample, and the Jaccard index, calculated using binarized data that represent the presence/absence of each OTU. The results were analyzed via PERMANOVA with 10,000 permutation simulations. Data regarding non-cyanobacterial OTUs were excluded, as were data from sampling locations that contained only non-cyanobacterial OTUs.

R software, version 3.6.3 (R Core Team 2020), was used for all statistical analyses; the package “car” (Fox and Weisberg 2019) was used for analysis of deviance and the package “vegan” (Oksanen et al. 2020) was used for PERMANOVA.

## Results

### Acetylene reduction rates and colonization rates in moss mats of *H*.* splendens* and* P*.* schreberi*

The acetylene reduction rate (mean ± standard deviation) of *H*. *splendens* was 0.68 ± 0.72 μmol g^−1^ day^−1^ on fallen logs and 0.27 ± 0.39 μmol g^−1^ day^−1^ on the ground surface; the corresponding values for *P*. *schreberi* were 0.20 ± 0.31 μmol g^−1^ day^−1^ and 0.10 ± 0.14 μmol g^−1^ day^−1^.

**Fig. 1:**
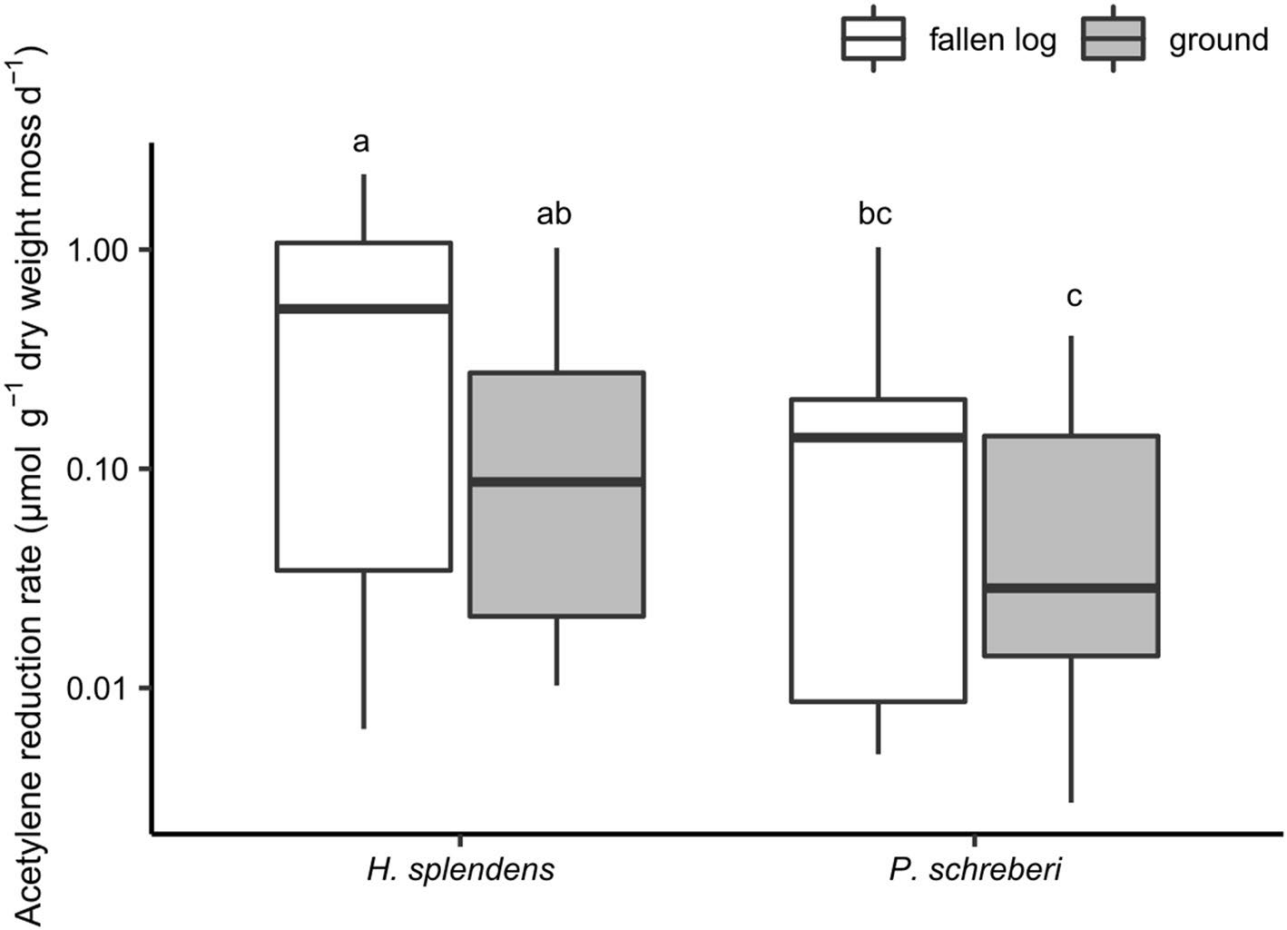
Acetylene reduction rates associated with *H*. *splendens* and *P*. *schreberi* (*n* = 10). Horizontal line, median; lower end of box, first quartile (25%); upper end of box, third quartile (75%). Ends of the whiskers represent the maximum and minimum values within 1.5 times the length of the box. Different letters indicate significant differences between log-transformed values (post hoc Tukey’s honestly significant difference test, *P* < 0.05)

**Fig. 2:**
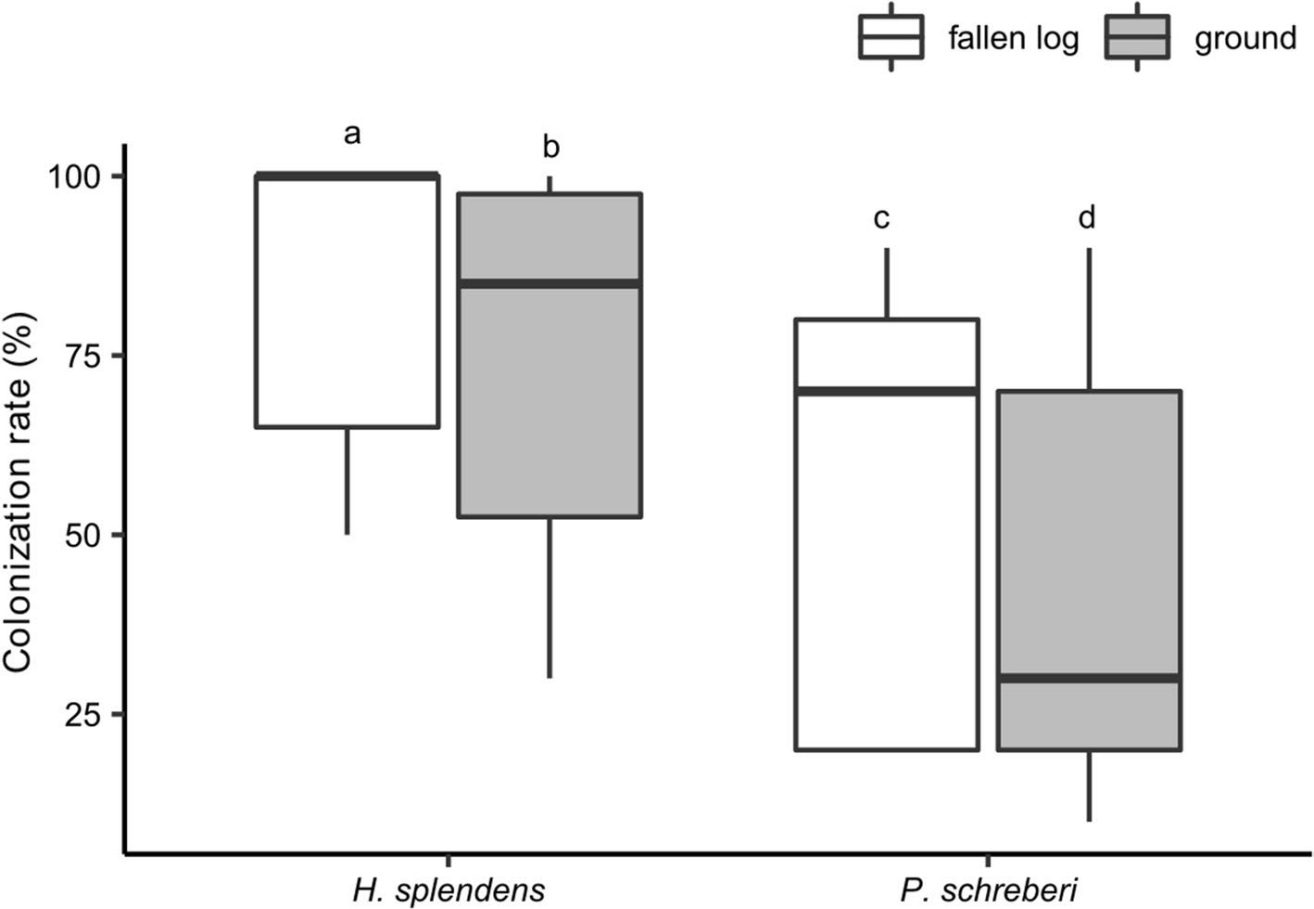
Cyanobacterial colonization rates of *H*. *splendens* and *P*. *schreberi* (*n* = 10). In the boxplot, the horizontal line shows the median, the lower end of the box shows the first quartile (25%), and the upper end of the box shows the third quartile (75%). The ends of the whiskers are the maximum and minimum values within 1.5 times the length of the box. Different letters in the figure indicate significant differences (Bonferroni-corrected Wilcoxon signed-rank test, *P* < 0.01)

**Fig. 3:**
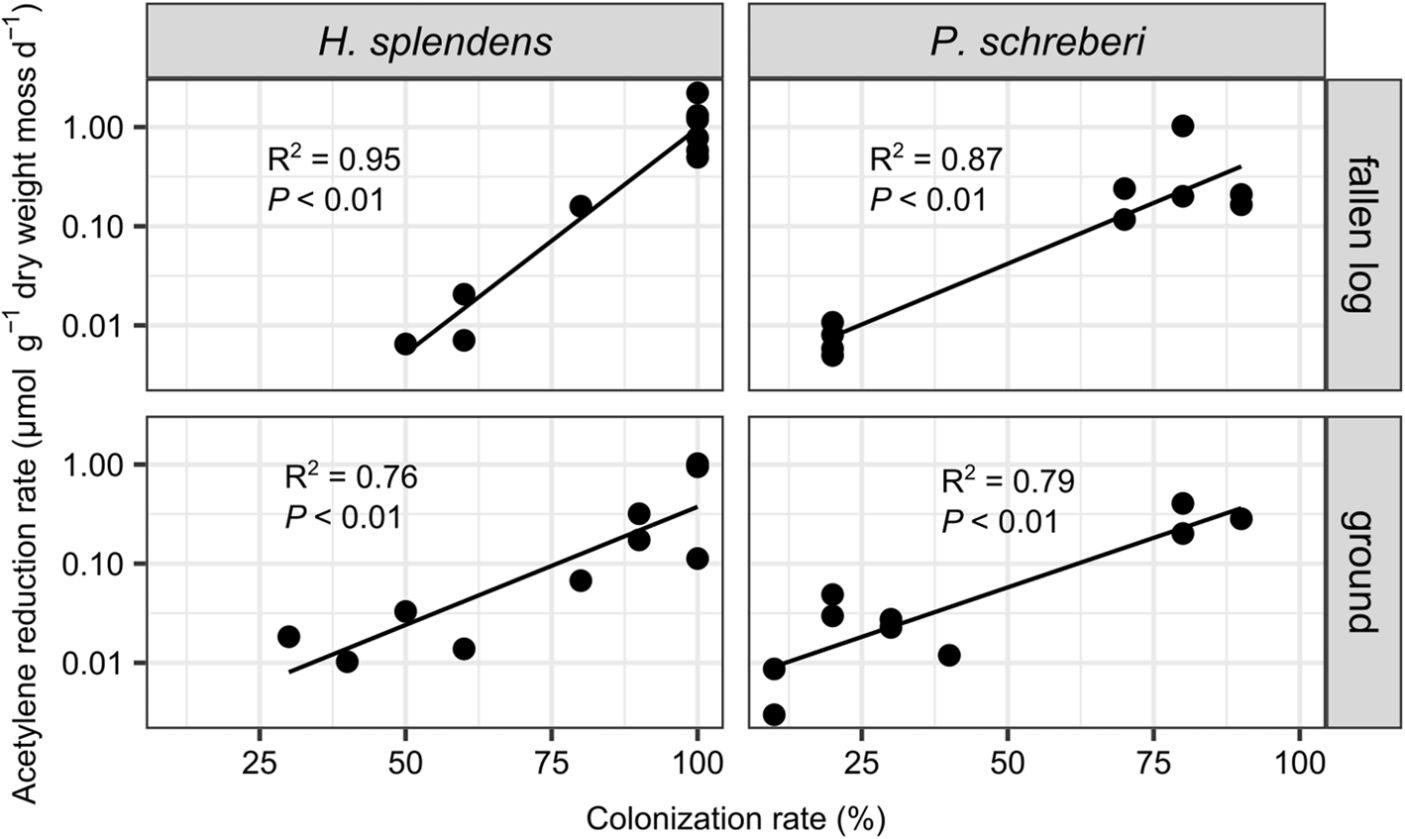
Relationship between cyanobacterial colonization rate and the logarithmic conversion value of the acetylene reduction rate (*n* = 10). Figures on the left show the relationship in *H. splendens*, while figures on the right show the relationship in *P. schreberi*. The upper figures show the relationship in mosses on fallen logs, while the lower figures show the relationship in mosses on the ground. The colonization rate was calculated as the proportion of shoots with cyanobacterial colonization among the 10 included shoots

There was strong evidence for differences in log-transformed acetylene reduction rates between *H*. *splendens* and *P*. *schreberi* (ANOVA: *F* = 11.0, *P* = 0.002), and a trend towards differences in log-transformed acetylene reduction rates associated with mosses collected from fallen logs and the ground surface (*F* = 3.9, *P* = 0.053). The acetylene reduction rate associated with *H*. *splendens* was higher than *P*. *schreberi* (Fig. 1). The acetylene reduction rate associated with moss samples collected from fallen logs tended to be higher than that of samples collected from the ground surface (Fig. 1). The cyanobacterial colonization rate of mosses was higher on *H*. *splendens* than on *P*. *schreberi* and on fallen logs than on the ground surface (Fig. 2). The log-transformed acetylene reduction rate associated with each moss species in each growth substrate (fallen logs/ground) was strongly positively correlated with the cyanobacterial colonization rate (Fig. 3).

### Environmental factors that correlate with the rate of acetylene reduction and cyanobacterial colonization

There was moderate evidence that the total nitrogen concentration in moss shoots was correlated with the acetylene reduction rates associated with both *H*. *splendens* and *P*. *schreberi* (analysis of deviance, type II test: *F* = 8.5 and 8.2, respectively; *P* < 0.05) and it correlated negatively with the acetylene reduction rate (Table 1). For *H*. *splendens*, there was moderate evidence that the acetylene reduction rate varied with moss growth substrate (analysis of deviance, type II test: *F* = 5.0, *P* = 0.041); *H*. *splendens* collected from fallen logs had higher acetylene reduction rate than those collected from the ground surface (Table 1). For both *H*. *splendens* and *P*. *schreberi*, there was no evidence that the acetylene reduction rates were not correlated with openness (analysis of deviance, type II test: *F* = 1.2 and 0.3, respectively; *P* > 0.1).

**Table 1:** Correlation of environmental factors with the acetylene reduction rate (GLM using a gamma distribution and log link)

Predictor	*H. splendens*		*P. schreberi*	
	Coefficient	Standard error	Coefficient	Standard error
Intercept	1.373	3.297	6.919	3.888
Habitat (ground)	− 1.248*	0.481	− 1.065	0.586
Openness	0.199	0.145	− 0.115	0.172
*N* concentration of moss shoots	− 5.637*	1.921	− 7.612*	2.272

*Indicates significant predictors in analysis of deviance (*P* < 0.05)

There was strong evidence that the total nitrogen concentration in moss shoots of *H*. *splendens* and *P*. *schreberi* was correlated with the cyanobacterial colonization rate (analysis of deviance, type II test: *F* = 9.5 and 10.8, respectively; *P* < 0.01); it was negatively correlated with the cyanobacterial colonization rate (Table 2). For *H*. *splendens*, there was strong evidence that canopy openness was correlated with cyanobacterial colonization (analysis of deviance, type II test: *F* = 9.1, *P* = 0.008); it was positively correlated with the cyanobacterial colonization rate (Table 2). By contrast, for *P*. *schreberi*, there was no evidence that openness was correlated with the colonization rate, but the colonization rate tended to differ depending on the growth substrate (analysis of deviance, type II test: *F* = 4.2, *P* = 0.057), with samples from fallen logs tending to have higher colonization rates than those from the ground surface (Table 2).

**Table 2:** Correlation of environmental factors with the cyanobacterial colonization rate (GLM using a quasi-binomial distribution and logit link)

Predictor	*H. splendens*		*P. schreberi*	
	Coefficient	Standard error	Coefficient	Standard error
Intercept	− 5.234	3.039	6.093	4.088
Habitat (ground)	− 0.432	0.413	− 1.062	0.533
Openness	1.116*	0.293	0.035	0.179
*N* concentration of moss shoots	− 11.362*	2.823	− 6.986*	2.45

*Indicates significant predictors in analysis of deviance (*P* < 0.05)

### Community composition of cyanobacteria living on *H. splendens* and* P. schreberi*

**Fig. 4:**
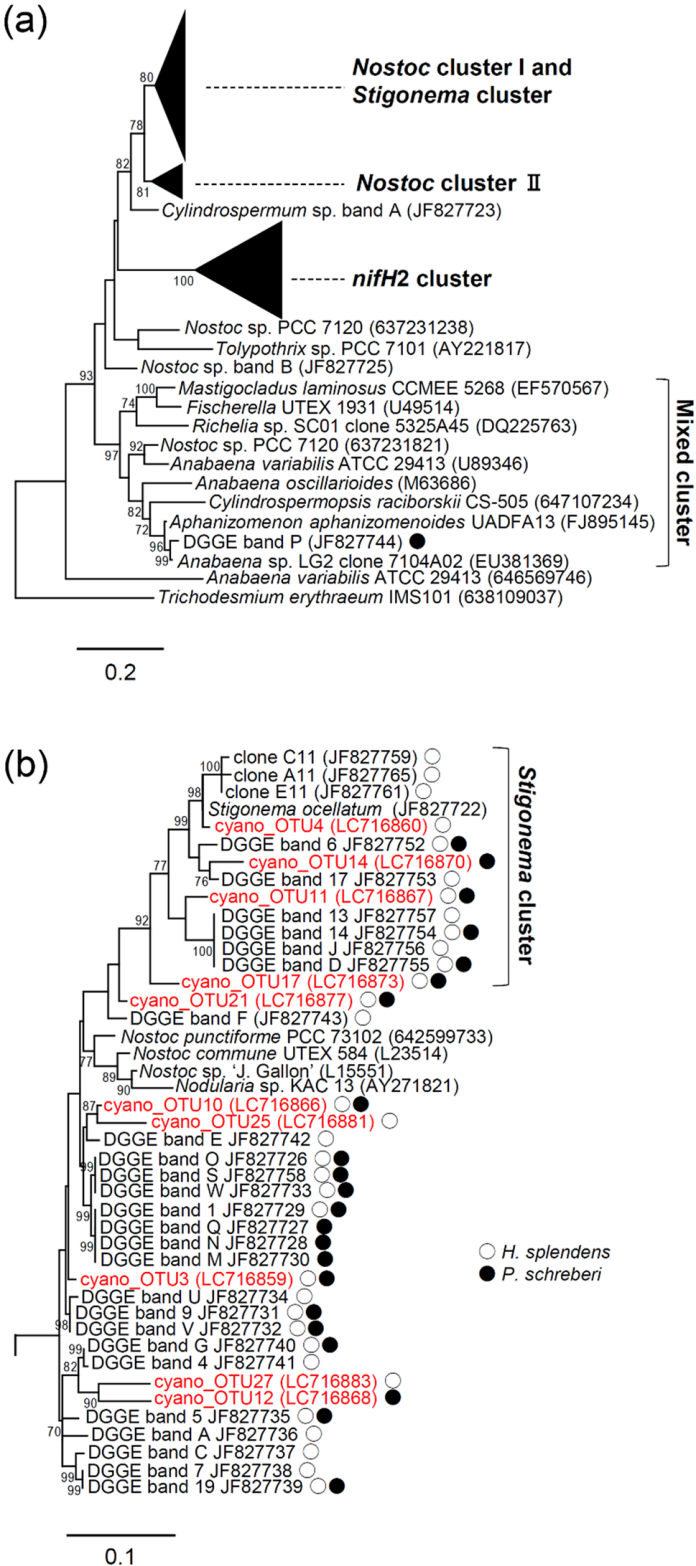

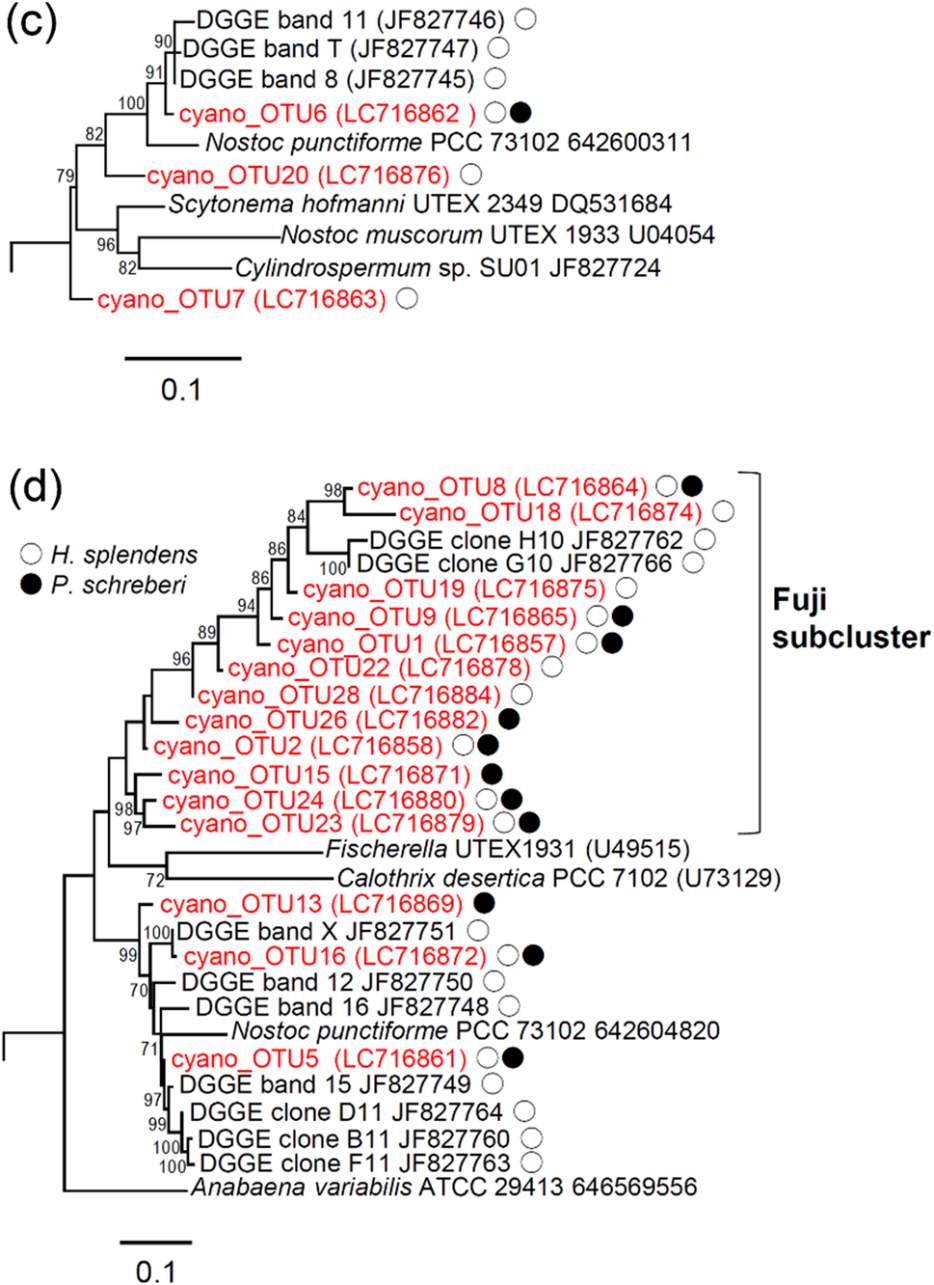
Phylogenetic tree of **a** all clusters, **b** *Nostoc* cluster I and *Stigonema* cluster, **c** *Nostoc* cluster II, and **d** *nifH*2 cluster based on the partial *nifH* sequence of cyanobacteria found on *H. splendens* and *P. schreberi*, determined using the maximum likelihood method. The numbers represent ultrafast bootstrap values. Reference sequences were obtained from GenBank and Integrated Microbial Genomes. Sequences labeled cyano_OTU are from this study; other sequences are from the phylogenetic tree in the work by Ininbergs et al. (2011). White and black circles indicate OTUs detected in *H. splendens* and *P. schreberi*, respectively

Of the 43 OTUs, 28 were classified as cyanobacteria and the remaining 15 were classified as non-cyanobacteria, based on the BLAST results. In the phylogenetic tree of cyanobacterial OTUs, *Nostoc* cluster I, identified by Ininbergs et al. (2011), was not monophyletic; it was located in the same clade as the *Stigonema* cluster (Fig. 4a, b). Of the 28 cyanobacteria OTUs, 10 were classified as *Nostoc* cluster I and *Stigonema* cluster, 3 were classified as *Nostoc* cluster II, and 15 were classified as *nifH*2 cluster (Fig.  b–d). In the *nifH*2 cluster, 12 OTUs were classified into a subcluster that we designated as the “Fuji subcluster” (Fig. 4d). Of the 28 cyanobacterial OTUs, 23 were found on *H. splendens*, 19 were found on *P. schreberi*, and 14 were found on both species (Fig. 4b–d).

Among the non-cyanobacterial OTUs, the most homologous (> 90%) bacterial sequences in BLAST were *Bradyrhizobium* (4 OTUs), *Methylocella* (2 OTUs), *Methyloferula* (2 OTUs), and unknown (7 OTUs).

When the Bray–Curtis index was used as the β-diversity index, PERMANOVA identified moss species and sampling location as factors that significantly affected the community composition of cyanobacterial OTUs; however, when the Jaccard index was used as the β-diversity index, only the sampling location was significant (Table 3).

**Table 3:** Effects of moss species, habitat, and sampling locations on cyanobacterial communities

Factor	PERMANOVA (Bray–Curtis)				PERMANOVA (Jaccard)			
	*Df*	*F*	*R*^*2*^	*p*	Df	*F*	*R*^*2*^	*p*
Moss species	1	2.61	0.062	0.013*	1	1.42	0.036	0.158
habitat (log/ground)	1	1.101	0.026	0.348	1	1.252	0.032	0.237
Sampling location	9	1.515	0.322	0.015*	9	1.326	0.301	0.037*
Residual	25		0.59		25		0.631	
Total	36		1		36		1	

Df means degree of freedom

*Indicates significant *p* values (*p* < 0.05)

## Discussion

### Acetylene reduction rate in subalpine moss carpets of *H*.* splendens* and* P*.* schreberi*

In our study, the acetylene reduction rate of *H*. *splendens* tended to be higher than that of *P*. *schreberi*. Zackrisson et al. (2009) reported that *H*. *splendens* had a lower sensitivity of associated acetylene reduction rate per unit area to nitrogen deposition compared with *P*. *schreberi*; when *N* availability was low, the acetylene reduction rate was inferior to *P*. *schreberi*, but when N availability was high, the acetylene reduction rate was slightly higher than *P*. *schreberi*. Jean et al. (2020) reported that *H*. *splendens* had a higher rate of nitrogen fixation per dry weight of moss and a higher abundance of genus *Nostoc* compared to *P*. *schreberi*. They speculated that *P*. *schreberi* had a lower nitrogen requirement than *H*. *splendens* since *P*. *schreberi* could tolerate a wider range of habitats and lower nutrient availability. In the pine forests at the northern foot of Mt. Fuji (8 km from the study site), nitrogen deposition is approximately 10 kg N ha^−1^ year^−1^, which is regarded as nitrogen saturation in forest ecosystems (Matsumoto et al. 2020). Furthermore, the study area is surrounded by a busy mountain road (Mt. Fuji Toll Road Fuji Subaru Line), and air pollution by NO and NO_2_ has been confirmed in the forest near the road (Wada et al. 2010). *P*. *schreberi* around high-traffic roads have lower acetylene reduction rates than *P*. *schreberi* around low-traffic roads (Ackermann et al. 2012). In addition, cyanobacterial colonization of *P*. *schreberi* in this study tended to be lower than that of *H*. *splendens*, suggesting *P*. *schreberi* may not actively attract cyanobacteria due to increased N availability caused by air pollution around Mt. Fuji subalpine forest, and as a result, the acetylene reduction rate may be lower than that of *H*. *splendens*. However, the nitrogen fixation rate of *H*. *splendens* and *P*. *schreberi* is seasonally and annually variable (Warshan et al. 2016; Jean et al. 2018), and our data were obtained only in September 2016.

The dry weight (mean ± standard deviation) per unit area of mosses in the study area was 141 ± 69 g m^−2^ for *H*. *splendens* and 90 ± 28 g m^−2^ for *P*. *schreberi* (Nakamura 1984). The mean (± standard deviation) acetylene reduction rates per unit land area calculated using these values were 95.3 ± 101.5 μmol m^−2^ day^−1^ on fallen logs and 38.2 ± 54.6 μmol m^−2^ day^−1^ on the ground surface for *H*. *splendens*; corresponding values for *P*. *schreberi* were 17.9 ± 27.5 μmol m^−2^ day^−1^ and 9.4 ± 12.78 μmol m^−2^ day^−1^, respectively. Using a moss growth period of 200 days and a conversion ratio of 3 mol acetylene molecules to 1 mol nitrogen molecules (DeLuca et al. 2002; Zackrisson et al. 2009), the nitrogen fixation associated with *H*. *splendens* and *P*. *schreberi* at the surface was calculated to be 0.89 ± 1.3 kg N ha^−1^ year^−1^. This value is one-eighth to one-half of the nitrogen fixation reported in late successional and less susceptible to nitrogen deposition boreal forests in Northern Europe and in Alaskan boreal spruce forest (Zackrisson et al. 2004, 2009; DeLuca et al. 2007; Jean et al. 2018). There is a clear latitudinal gradient in nitrogen fixation per area and per moss dry weight associated with *H*. *splendens* and *P*. *schreberi* from northern to southern Fennoscandia, and it is speculated that nitrogen fixation is greatly limited by anthropogenic nitrogen deposition in boreal forests of southern Fennoscandia (Zackrisson et al. 2009; Salemaa et al. 2019). As mentioned above, there is a busy mountain road around the study site on Mt. Fuji and high nitrogen deposition near the study site has been reported; such high anthropogenic nitrogen supply may have resulted in inferior nitrogen fixation associated with feather moss on the ground surface in the subalpine forest of Mt. Fuji compared to the boreal forest in the northern Fennoscandian region. Light availability on the forest floor may also explain differences in nitrogen fixation on the moss forest floor in subalpine forests of Mt. Fuji and boreal forests of northern Fennoscandia and Alaska. The basal area of the study site is up to threefold that of boreal forests with feathermoss-associated nitrogen fixation (DeLuca et al. 2007; Melvin et al. 2015; Jean et al. 2018), and canopy coverage, calculated from the openness of the sampled sites, is as high as in Alaskan paper birch forests with lower nitrogen fixation rates associated with feather mosses (Jean et al. 2018). Canopy cover is negatively correlated with the abundance of Nostocaceae in feather mosses, resulting in reduced nitrogen fixation per moss dry weight (Jean et al. 2020). Therefore, cyanobacterial abundance and nitrogen fixation may be lower in the subalpine forests of Mt. Fuji due to low light availability caused by high forest canopy cover compared to boreal forests in northern Fennoscandia and Alaska. However, the nitrogen fixation rate was based on acetylene reduction measured in the laboratory 12 days after sample collection, values for moss biomass in 1984 were used, and the conversion ratio between acetylene and nitrogen molecules can vary with moss species and time (Saiz et al. 2019).

There was a strong positive correlation between the acetylene reduction rate and the cyanobacterial colonization rate in both *H*. *splendens* and *P*. *schreberi*, consistent with findings in previous studies (DeLuca et al. 2007; Stuart et al. 2021a; Liu and Rousk 2022; Renaudin et al. 2022). While positive correlations between nitrogen fixation and relative abundances of bacteria other than cyanobacteria have been reported for some moss species (Holland-Moritz et al. 2021), the strong correlation between the acetylene reduction rate and the rate of cyanobacterial colonization in this study suggests that cyanobacteria contribute to nitrogen fixation on moss carpets in subalpine forest of Mt. Fuji. However, because nitrogen-fixing bacteria other than cyanobacteria carrying the *nifH* gene were also identified, and nitrogen-fixing activity was observed even in samples with low cyanobacterial colonization rates (10%), the possibility that nitrogen-fixing bacteria other than cyanobacteria contributed to the acetylene reduction rates cannot be excluded.

### Environmental factors that correlate with the rate of acetylene reduction and cyanobacterial colonization

In this study, among the analyzed factors (i.e., growth substrate [fallen logs or ground surface], openness, and total nitrogen concentration in moss shoots), the strongest correlation with the acetylene reduction rate and colonization rate was mediated by the total nitrogen concentration in moss shoots, which was negatively correlated with the acetylene reduction rate and colonization rate in both moss species. Mosses in nitrogen-poor environments release chemical signals that promote the differentiation of cyanobacteria into hormogonia, a motile form that enhances the colonization of moss shoots (Bay et al. 2013). This may explain our finding of a negative correlation between the cyanobacterial colonization rate and the total nitrogen concentration in moss shoots. A study of seven forest sites in Finland identified positive correlations between nitrogen fixation rates per moss dry weight and the C/N ratios of moss shoots of *H*. *splendens* and *P*. *schreberi* (Leppänen et al. 2013). Because our results were obtained from samples in a 1-ha forest area, the negative correlation between the acetylene reduction rate and the total nitrogen concentration in moss shoots strongly suggests that cyanobacterial nitrogen fixation activity has high sensitivity to the nitrogen requirement of the host moss.

Light availability was positively correlated with the abundance of Nostocaceae associated with feather mosses (Jean et al. 2020), and the relationship between the cyanobacterial colonization rate and the canopy openness in *H*. *splendens* in this study is consistent with a prior report. By contrast, there was no correlation between the acetylene reduction rate and openness. Jean et al. (2020) reported that in both *H*. *splendens* and *P*. *schreberi*, canopy cover has a negative effect on moss-related nitrogen fixation directly or through relative abundance of Nostocaceae. In manipulation experiments, light availability was positively correlated with nitrogen fixation except at temperatures exceeding 30 °C (Gundale et al. 2012a; Sorensen et al. 2012). This discrepancy may be due to the fact that the mosses were incubated under the same light conditions in the laboratory during the acetylene reduction assay. It has been suggested that the tree canopy changes not only light availability but also the amount and form of nitrogen in rainfall, affecting nitrogen fixation associated with feathermoss on the forest floor (Salemaa et al. 2019). Because tree canopy reduces total N deposition and increases the proportion of dissolved organic nitrogen (DON) in through fall, sites with greater openness have more inorganic nitrogen, which is likely to negatively affect nitrogen fixation associated with feather moss, compared to sites with less openness (Salemaa et al. 2019).

In the GLM, acetylene reduction rates associated with *H*. *splendens* tended to be higher in samples collected from fallen logs than from the ground surface. In addition, although not significant by analysis of deviance, multiple comparisons showed that the cyanobacterial colonization rates of *H*. *splendens* and *P*. *schreberi* tended to be higher in samples collected from fallen logs than from the ground surface. The reasons for these findings are unclear, but microenvironmental differences such as light availability, leaf litter accumulation (Jean et al. 2020), and nutrient availability may have contributed. *H*. *splendens* and *P*. *schreberi* appear when logs are considerably decomposed (Nakamura 1987). Fallen logs, as coarse woody debris, can be a source of nutrients such as phosphorus as they decompose (Laiho and Prescott 2004; Brais et al. 2006). Cases of phosphorus promoting cyanobacterial biomass (Renaudin et al. 2022) and acetylene reduction (Zackrisson et al. 2004) have been reported. This result may be due to the greater P supply from decayed fallen logs than from the ground surface.

### Community composition of cyanobacteria on *H. splendens* and* P. schreberi*

Among the five cyanobacterial clusters identified by Ininbergs et al. (2011), OTUs that belonged to four clusters (all but the mixed cluster) were detected in this study. To our knowledge, this is the first report that major cyanobacterial clusters common to the boreal forests of Northern Europe are also present in Japanese subalpine forests. These four cyanobacterial clusters are widely distributed in the Northern Hemisphere, along with *H. splendens* and *P. schreberi*. However, in the present study, the largest number of OTUs belonged to the *nifH*2 cluster; 12 of 15 OTUs in that cluster belonged to the “Fuji subcluster,” which is rare in the boreal forests of Northern Europe (Ininbergs et al. 2011). These results suggest global differences in terms of cyanobacterial communities between boreal forests and subalpine forests in the temperate zone.

PERMANOVA showed no effect of moss species on cyanobacterial community composition, according to presence/absence data (i.e., using the Jaccard index); however, a difference was detected when abundance data (i.e., using the Bray–Curtis index) were used. Therefore, although the basic cyanobacterial community composition was similar for the two moss species, there may be subtle differences in the composition preferred by each moss species. Differences in host moss phylogenetic distance and moss microbiome composition are correlated (Holland-Moritz et al. 2021). The two mosses in this study belong to the same family; they have a close phylogenetic relationship. Notably, we found that half of the identified OTUs of cyanobacteria were present in both *H. splendens* and *P. schreberi*, suggesting that the two mosses are intermixed and thus share some strains of cyanobacteria for nitrogen fixation.

In conclusion, this study revealed the following: (1) In a subalpine forest on Mt. Fuji, cyanobacteria colonized and fixed nitrogen in feather mosses, and *H*. *splendens* was superior to *P*. *schreberi* in acetylene reduction rate in September. (2) Analysis of *nifH* of cyanobacteria on feather mosses in a subalpine forest of Mt. Fuji revealed a community composed of four cyanobacterial clusters that are common to boreal forests. (3) Even within a seemingly homogeneous forest stand, the acetylene reduction rate associated with feather mosses differed depending on the growth substrate and the total nitrogen concentration of the mosses, and had a particularly strong relationship with the latter.

To our knowledge, this is the first phylogenetic analysis of cyanobacteria on feather mosses, and specifically on *H. splendens* and *P. schreberi*, in East Asia. The data obtained from this subalpine forest, which is spatially remote from boreal forests, will contribute to a better understanding of consistency of responses to environmental factors and cyanobacterial community structure in moss–cyanobacteria associations.

## Data Availability

The datasets used in this study are available from the corresponding author on reasonable request. Sequences of each OTU are registered in the DDBJ database (accession numbers LC716857–LC716899).
